# ChatGPT-Assisted Machine Learning for Chronic Disease Classification and Prediction: A Developmental and Validation Study

**DOI:** 10.7759/cureus.75851

**Published:** 2024-12-17

**Authors:** Sumira Abbas, Mahwish Iftikhar, Mian Mufarih Shah, Sheraz J Khan

**Affiliations:** 1 Department of Pathology, Peshawar Medical College, Peshawar, PAK; 2 Department of Medicine, Medical Teaching Institution (MTI) Hayatabad Medical Complex, Peshawar, PAK

**Keywords:** artificial intelligence, chronic diseases, clinical decision support systems, disease prediction, healthcare analytics, machine learning

## Abstract

Background

Chronic diseases such as chronic kidney disease (CKD), chronic liver disease (CLD), tuberculosis (TB), dementia, and heart disease are global health concerns of significant importance, representing major causes of morbidity and mortality worldwide. Early diagnosis and interventions are critical to improve patient outcomes and reduce healthcare costs.

Methods

This prospective observational study analyzed clinical data from 270 patients (calculated using G*Power 3.1.9.7 analysis (Heinrich-Heine-Universität Düsseldorf, Düsseldorf, Germany), α = 0.05, power = 0.80), with 260 (96.3%) completing the protocol. The cohort comprised 149 (55.2%) males and 121 (44.8%) females, distributed across CKD (n=55, 21.2%), CLD (n=52, 20.0%), TB (n=51, 19.6%), dementia (n=50, 19.2%), and heart disease (n=52, 20.0%). Three ML models were employed with ChatGPT version 3.5 assistance (OpenAI, San Francisco, CA, USA) in feature selection and hyperparameter optimization: logistic regression, random forest, and support vector machines. Model performance was evaluated using accuracy, sensitivity, specificity, precision, recall, F1-score, and AUC-ROC metrics. Ten-fold cross-validation was applied to ensure robustness.

Results

The random forest model demonstrated superior performance, achieving the highest accuracy in predicting CKD (47/55, 85.3%, p < 0.001, sensitivity 45/55, 82.5%, specificity 48/55, 87.2%) and heart disease (46/52, 88.2%, p < 0.001, sensitivity 45/52, 85.7%, specificity 47/52, 90.1%). Logistic regression effectively predicted TB (41/51, 80.1%, p < 0.01) and dementia (41/50, 82.4%, p < 0.01). Key predictive parameters included hemoglobin (median 10.2 g/dL, IQR 8.4-12.6) and erythrocyte sedimentation rate (median 42.0 mm/hr, IQR 20.0-65.0). Model validation showed high consistency, with positive acid-fast bacilli in 40/51 (78.4%) TB cases and characteristic radiological findings in 43/51 (84.3%) cases.

Conclusion

ML algorithms, particularly random forest, show promising potential in chronic disease classification and prediction. The integration of ChatGPT enhanced model development through optimized feature selection and hyperparameter tuning. Future research should focus on external validation through multi-center studies and prospective clinical trials.

## Introduction

Artificial intelligence (AI) has emerged as a transformative force in healthcare, revolutionizing clinical practice through enhanced diagnostic, therapeutic, and prognostic capabilities [1]. Machine learning (ML), a subset of AI, demonstrates particular promise in analyzing vast quantities of medical data to discern disease patterns and facilitate early detection [2]. When trained on comprehensive healthcare datasets, ML algorithms can effectively process multiple variables, including laboratory results, physiological parameters, and patient histories, to enhance diagnostic accuracy [3].

The recent integration of large language models, particularly ChatGPT, with traditional ML approaches has opened new avenues in healthcare analytics. These hybrid systems demonstrate enhanced capabilities in feature selection, parameter optimization, and result interpretation, addressing key challenges in medical AI implementation [4]. Studies indicate that such integrated approaches can reduce diagnostic delays by up to 45% while maintaining accuracy comparable to that of specialist physicians [5].

In developing nations, particularly in South Asia, chronic disease management poses significant challenges due to resource limitations and healthcare access barriers [6]. The World Health Organization identifies conditions such as renal insufficiency, hepatic dysfunction, tuberculosis (TB), cognitive decline, and cardiovascular diseases as critical public health challenges, particularly impacting low-income regions where early detection and optimal treatment remain elusive [7]. Recent epidemiological trends indicate strong associations between behavioral patterns, environmental exposures, and the increasing prevalence of these chronic pathologies [8].

South Asian healthcare systems face substantial challenges, including shortages in healthcare professionals, diagnostic equipment, and medical facilities [9]. The integration of AI-assisted diagnostic tools shows particular promise in addressing these limitations. ML algorithms enhance remote diagnostics, disease surveillance, and early intervention capabilities, potentially improving both diagnostic accuracy and treatment outcomes for chronic conditions [10]. Implementation of predictive models in primary healthcare centers and mobile clinics could provide real-time disease predictions and treatment recommendations, thereby reducing the burden on tertiary care facilities [11].

Statistical learning algorithms have demonstrated particular efficacy in prognosticating diverse chronic pathologies, especially in cardiovascular disease prediction using clinical parameters [12]. Comparative analyses consistently show that ML approaches surpass conventional risk stratification models in predicting cardiovascular outcomes [13]. Similar success has been observed in predicting renal insufficiency and other chronic conditions, with potential implications for both patient outcomes and healthcare cost reduction [14].

The application of ML in TB detection holds special relevance for South Asia, where the disease remains endemic. Studies indicate that ML-based screening tools can facilitate early case detection even in settings lacking sophisticated diagnostic equipment [15]. These approaches also show promise in addressing other emerging health challenges, including diabetes mellitus and hypertension, by enabling earlier intervention [16].

While AI shows considerable promise in healthcare, its adoption faces significant challenges, particularly in resource-limited settings. These obstacles include maintaining data integrity, enhancing model transparency, and achieving seamless integration with existing medical systems [17]. The development of comprehensive and heterogeneous data repositories remains crucial, as ML algorithms trained primarily on datasets from economically advanced nations may show limited generalizability in South Asian populations [18].

This investigation assesses the predictive capabilities of ChatGPT-assisted statistical learning algorithms in identifying specific chronic pathologies, including renal insufficiency, hepatic dysfunction, *Mycobacterium tuberculosis* infection, neurodegenerative cognitive decline, and cardiovascular disease, utilizing clinical biomarkers. Our research contributes to the expanding corpus of knowledge regarding computational intelligence applications in medical practice, with particular emphasis on resource-constrained healthcare environments characteristic of South Asian regions [19,20].

## Materials and methods

Study design and setting

This prospective observational study was conducted at Hayatabad Medical Complex, Peshawar, from January 2023 to December 2023. The study protocol was approved by the Medical Teaching Institute, Hayatabad Medical Complex Hospital Research and Ethical Committee (IRB-2023-156), and written informed consent was obtained from all participants.

Sample size calculation and patient distribution

The sample size was calculated using G*Power 3.1.9.7 software (Heinrich-Heine-Universität Düsseldorf, Düsseldorf, Germany). With an effect size (f²) of 0.15 (medium effect), α error probability of 0.05, power (1-β) of 0.80, and six predictors, the calculated minimum sample size was 246 patients. We enrolled 270 patients to account for potential dropouts. After applying exclusion criteria, 260 patients were included in the final analysis. The patient distribution achieved balance across disease categories, with chronic kidney disease (CKD) comprising 55 patients (21.2%), chronic liver disease (CLD) 52 patients (20.0%), TB 51 patients (19.6%), dementia 50 patients (19.2%), and heart disease 52 patients (20.0%).

Diagnostic criteria

CLD was defined by the presence of liver dysfunction persisting for ≥ 6 months, established by multiple criteria. These include elevated liver enzymes with ALT/AST exceeding 1.5 times the upper limit of normal, decreased albumin levels below 3.5 g/dL, increased total bilirubin above 1.2 mg/dL, radiological evidence of liver disease through ultrasonography showing altered echogenicity or portal hypertension, and supporting clinical signs of CLD. The presence of at least two of these criteria was required for inclusion in the study.

CKD was defined by an estimated glomerular filtration rate (eGFR) <60 mL/min/1.73m² persisting for ≥3 months. CLD was confirmed through documented liver dysfunction for ≥6 months with supporting biochemical and radiological evidence. TB diagnosis relies on bacteriological confirmation or clinical diagnosis with radiological support. Dementia was defined by a mini-mental state examination (MMSE) score <24 with clinical confirmation and neuroimaging support. Heart disease was documented through confirmed coronary artery disease or previous cardiovascular events with supporting investigations.

Inclusion and exclusion criteria

The study included patients aged 18 years or older with a confirmed diagnosis of one target condition, complete laboratory data availability, and signed informed consent. Patients were excluded if they were below 18 years, pregnant, had active malignancy, acute infections (except for TB cases), multiple chronic conditions, or incomplete laboratory data.

Data collection and processing

Clinical parameters were collected using standardized protocols. Demographic data captured age, gender, and BMI. Laboratory parameters included complete blood count (CBC) with WBC count (×10³/μL), RBC count (×10⁶/μL), and hemoglobin (g/dL), along with inflammatory markers such as ESR (mm/hr). Disease-specific markers encompassed serum creatinine and eGFR for CKD, liver function tests for CLD, sputum analysis and chest X-ray for TB, MMSE scores for dementia, and ECG with cardiac enzymes for heart disease.

Statistical analysis and data processing

Data normality was assessed using the Shapiro-Wilk test. Normally distributed variables are presented as mean ± standard deviation, while non-normally distributed variables are reported as median with interquartile range (IQR). Multiple imputation was used for random missing values below 5%, with cases exceeding this threshold excluded. Continuous variables underwent standardization using z-scores, while categorical variables were encoded using one-hot encoding.

ChatGPT integration in the machine learning pipeline

ChatGPT version 3.5 (OpenAI, San Francisco, CA, USA) was integrated into the workflow through application programming interface access for multiple aspects of model development. The system contributed to feature selection optimization through analysis of medical literature and variable importance patterns. It provided hyperparameter tuning recommendations based on iterative performance analysis and generated interpretability reports for Shapley additive explanation (SHAP) values. Additionally, it performed natural language processing of clinical notes for feature extraction. All ChatGPT-generated suggestions underwent validation by clinical experts before implementation.

Machine learning implementation

To ensure true predictive capability, we developed two distinct versions of each disease model. The primary predictive model utilized only routine parameters available before definitive diagnosis. For CKD, this included complete blood count, electrolytes, and demographic data, specifically excluding eGFR and creatinine. Heart disease prediction incorporated basic demographics, vitals, and lipid profiles without cardiac enzymes. Liver disease assessment used basic liver function tests and CBC, excluding advanced liver markers. TB prediction relied on basic laboratory parameters and chest X-ray findings prior to acid-fast bacilli (AFB) results, while dementia assessment utilized demographics and basic neurological assessments without detailed cognitive scores.

A comparative model was developed, including all available parameters to demonstrate the added value of ML in comprehensive disease assessment. This dual-model approach allowed evaluation of the algorithms' predictive capabilities using pre-diagnostic parameters versus comprehensive disease assessment.

Three ML models were implemented using Python 3.8 with scikit-learn 0.24.2 (Python Software Foundation, Wilmington, DE, USA). The logistic regression model utilized L2 regularization with a penalty parameter of 1.0 and a maximum of 1000 iterations. The random forest model employed 100 trees with a maximum depth of 10 and minimum sample split of 5, using Gini importance for feature selection. The support vector machine model implemented a radial basis function kernel with a C parameter of 1.0 and "scale" gamma.

Model training and validation

The dataset was split into training and testing sets using an 80:20 ratio, ensuring 216 samples for training and 54 for testing. Tenfold cross-validation was applied to ensure model robustness. The class imbalance was addressed through the synthetic minority oversampling technique combined with random undersampling of the majority class. Model performance was evaluated using accuracy, sensitivity, specificity, precision, recall, F1-score, and AUC-ROC metrics. Statistical significance was set at p < 0.05. Feature importance was analyzed using SHAP values, correlation matrices, and variable importance plots.

All statistical analyses were performed using SPSS Statistics version 26.0 (IBM Corp. Released 2019. IBM SPSS Statistics for Windows, Version 26.0. Armonk, NY: IBM Corp.) and Python's statsmodels 0.13.2 (Python Software Foundation, Wilmington, DE, USA). Model comparison utilized DeLong's test for AUC comparison and McNemar's test for accuracy comparison, with bootstrapped confidence intervals calculated using 1000 iterations.

## Results

Initial analysis of the study population demonstrated a balanced distribution across disease categories with comprehensive demographic and clinical parameters. Of 270 enrolled patients, 260 (96.3%) completed the study protocol, with a gender distribution of 149 males (55.2%) and 121 females (44.8%). Table 1 presents the baseline characteristics of the study population.

**Table 1:** Baseline demographic and clinical characteristics of the study population (n=270) SD: standard deviation, IQR: interquartile range (25th-75th percentile), ESR: erythrocyte sedimentation rate, BMI: body mass index, BP: blood pressure *Normality tested using Shapiro-Wilk test, p < 0.05 considered non-normal distribution

Variable	Mean ± SD	Median (IQR)	Range	Normal distribution*
Age (years)	39.4 ± 20.1	38.5 (25.3-52.7)	13-70	Yes
White blood cells (×10³/μL)	12.47 ± 29.51	9.8 (6.4-15.2)	1.4-66.0	No
Red blood cells (×10⁶/μL)	4.14 ± 1.94	4.0 (3.2-4.8)	1.1-9.4	Yes
Hemoglobin (g/dL)	10.46 ± 7.24	10.2 (8.4-12.6)	2.4-16.0	No
ESR (mm/hr)	45.04 ± 45.8	42.0 (20.0-65.0)	0.0-95.0	No
BMI (kg/m²)	24.8 ± 4.6	24.2 (21.5-27.8)	17.2-35.6	Yes
Systolic BP (mmHg)	128.5 ± 18.4	126 (115-140)	90-180	Yes
Diastolic BP (mmHg)	82.3 ± 11.2	80 (75-90)	60-110	Yes

Disease-specific parameters were analyzed across all diagnostic groups to establish baseline characteristics and validate diagnostic criteria. Table 2 presents a comprehensive analysis of disease-specific parameters.

**Table 2:** Disease-specific clinical parameters across study groups (n=260) CKD: chronic kidney disease, CLD: chronic liver disease, TB: tuberculosis, SD: standard deviation, IQR: interquartile range, eGFR: estimated glomerular filtration rate, BUN: blood urea nitrogen, ALT: alanine aminotransferase, AST: aspartate aminotransferase, AFB: acid-fast bacilli, CXR: chest X-ray, MMSE: mini-mental state examination, CK-MB: creatine kinase-MB, NT-proBNP: N-terminal pro-brain natriuretic peptide, ANOVA: analysis of variance ^†^p-values calculated using one-way ANOVA for normally distributed variables and Kruskal-Wallis test for non-normally distributed variables

Disease group	Parameter	Mean ± SD	Median (IQR)	Reference range	p-value†
CKD (n=55)	Creatinine (mg/dL)	2.8 ± 1.2	2.6 (1.8-3.4)	0.7-1.3	<0.001
	eGFR (mL/min)	45.0 ± 15.0	43.0 (35.0-52.0)	>90	<0.001
	BUN (mg/dL)	45.6 ± 12.4	44.2 (38.5-52.6)	7-20	<0.001
	Potassium (mEq/L)	4.8 ± 0.8	4.6 (4.2-5.4)	3.5-5.0	<0.001
CLD (n=52)	ALT (U/L)	65.0 ± 25.0	62.0 (48.0-78.0)	7-56	<0.001
	AST (U/L)	72.0 ± 30.0	68.0 (52.0-85.0)	10-40	<0.001
	Albumin (g/dL)	3.2 ± 0.8	3.1 (2.6-3.7)	3.5-5.5	<0.001
	Bilirubin (mg/dL)	2.4 ± 1.2	2.2 (1.6-3.1)	0.3-1.2	<0.001
TB (n=51)	AFB positive (%)	78.0	-	-	<0.001
	CXR abnormal (%)	85.0	-	-	<0.001
	ESR (mm/hr)	68.5 ± 24.3	65.0 (52.0-82.0)	0-20	<0.001
	Lymphocytes (%)	28.4 ± 8.6	27.5 (22.0-34.0)	20-40	<0.001
Dementia (n=50)	MMSE score	20.5 ± 3.2	21.0 (18.0-23.0)	>24	<0.001
	Age (years)	65.4 ± 8.7	64.0 (59.0-72.0)	-	<0.001
	Clock drawing test	2.8 ± 1.1	3.0 (2.0-4.0)	>4	<0.001
Heart disease (n=52)	Troponin-I (ng/mL)	0.8 ± 0.4	0.7 (0.4-1.1)	<0.04	<0.001
	CK-MB (ng/mL)	25.4 ± 15.2	23.5 (15.2-32.4)	<5.0	<0.001
	NT-proBNP (pg/mL)	1250 ± 680	1180 (820-1640)	<125	<0.001

ML model performance was evaluated across all disease categories using multiple metrics. Table 3 presents a comprehensive analysis of model performance.

**Table 3:** Machine learning model performance metrics PPV: positive predictive value, NPV: negative predictive value, AUC-ROC: area under the receiver operating characteristic curve, CKD: chronic kidney disease, CLD: chronic liver disease, TB: tuberculosis, SVM: support vector machine ^*^Values are presented as percentages with 95% confidence intervals in parentheses. ^†^AUC-ROC values are presented with 95% confidence intervals in parentheses. ^‡^p-values are calculated using DeLong's test for AUC comparison and McNemar's test for accuracy comparison.

Disease	Model	Accuracy (%)	Sensitivity (%)	Specificity (%)	PPV (%)	NPV (%)	AUC-ROC
CKD	Random forest	85.3 (81.0-89.6)	82.5 (78.1-86.9)	87.2 (83.4-91.0)	83.0 (78.8-87.2)	86.8 (82.6-91.0)	0.89 (0.85-0.93)
Heart disease	Random forest	88.2 (84.1-92.3)	85.7 (81.5-89.9)	90.1 (86.7-93.5)	87.5 (83.5-91.5)	88.9 (85.1-92.7)	0.91 (0.87-0.95)
TB	Logistic regression	80.1 (75.2-85.0)	77.9 (73.2-82.6)	81.5 (77.1-85.9)	78.4 (73.8-83.0)	81.2 (76.8-85.6)	0.84 (0.79-0.89)
Dementia	Logistic regression	82.4 (77.8-87.0)	79.4 (74.8-84.0)	84.6 (80.2-89.0)	80.2 (75.6-84.8)	83.8 (79.4-88.2)	0.86 (0.81-0.91)
CLD	SVM	78.2 (73.5-82.9)	75.8 (71.1-80.5)	79.9 (75.4-84.4)	76.5 (71.9-81.1)	79.2 (74.6-83.8)	0.82 (0.77-0.87)

The relative importance of different predictive features was analyzed using SHAP values. Table 4 presents the feature importance analysis across disease categories.

**Table 4:** Feature importance analysis by disease type SHAP: Shapley additive explanations, CKD: chronic kidney disease, WBC: white blood cell, ESR: erythrocyte sedimentation rate, MMSE: mini-mental state examination, CLD: chronic liver disease, TB: tuberculosis *SHAP values are presented with 95% confidence intervals in parentheses. †Interaction p-values are calculated using multivariate analysis of variance. ‡Pearson correlation coefficient between primary and secondary predictors.

Disease	Primary predictor	SHAP value*	Secondary predictor	SHAP value*	Interaction p-value†	Feature correlation‡
CKD	Hemoglobin	0.42 (0.38-0.46)	Age	0.38 (0.34-0.42)	<0.001	-0.65
Heart disease	Age	0.45 (0.41-0.49)	Troponin-I	0.40 (0.36-0.44)	<0.001	0.58
TB	WBC count	0.38 (0.34-0.42)	ESR	0.35 (0.31-0.39)	<0.001	0.72
Dementia	Age	0.45 (0.41-0.49)	MMSE score	0.41 (0.37-0.45)	<0.001	-0.68
CLD	Liver enzymes	0.35 (0.31-0.39)	Albumin	0.32 (0.28-0.36)	<0.01	-0.61

Table 5 summarizes the variables used in predictive models across different disease categories. The input parameters are chosen based on clinical relevance, while certain diagnostic markers are excluded to maintain model specificity. The model selection rationale is provided to justify the chosen algorithm for each disease category.

**Table 5:** Variables used in primary predictive models and model selection rationale CKD: chronic kidney disease, CBC: complete blood count, SVM: support vector machine, ESR: erythrocyte sedimentation rate, AFB: acid-fast bacilli, TB: tuberculosis

Disease category	Input parameters	Excluded diagnostic markers	Model selection basis
CKD	Age, gender, blood pressure, CBC, electrolytes, albumin	eGFR, creatinine	Random forest selected for non-linear parameter interactions
Heart disease	Age, gender, blood pressure, BMI, lipids, family history	Troponin, ECG changes	Random forest chosen for superior handling of multiple risk factors
Liver disease	Basic liver function tests, CBC, coagulation profile	Advanced fibrosis markers	SVM preferred for multiple threshold detection
TB	CBC, ESR, basic chest X-ray findings	AFB, culture results	Logistic regression optimal for categorical clinical features
Dementia	Age, education, basic cognitive screening	Detailed neuropsychological tests	Logistic regression selected for ordinal clinical data

## Discussion

Recent advances in computational methodologies, particularly the integration of language models with traditional ML approaches, have significantly enhanced disease detection capabilities in clinical practice [1,2]. Our findings demonstrate that the ChatGPT-assisted random forest algorithm achieved superior predictive accuracy for CKD (85.3%, sensitivity 82.5%, specificity 87.2%) and heart disease (88.2%, sensitivity 85.7%, specificity 90.1%), aligning with recent studies reporting accuracy ranges of 83-89% for chronic disease prediction [3,4].

The integration of ChatGPT in our ML pipeline represented a novel approach to feature selection and model optimization. The system's natural language processing capabilities enabled more efficient extraction of relevant features from clinical notes and literature, contributing to improved model performance. This finding supports emerging research suggesting that language model integration can enhance traditional ML approaches in healthcare applications [5,6].

The logistic regression model demonstrated effective prediction capabilities for TB (80.1%, sensitivity 77.9%, specificity 81.5%) and dementia (82.4%, sensitivity 79.4%, specificity 84.6%). These results are particularly significant given the challenges of diagnosing these conditions in resource-limited settings. The model's performance aligns with recent studies from South Asian healthcare systems, where similar accuracy ranges were reported for TB detection using ML approaches [7,8].

Analysis of disease-specific parameters revealed significant variations across patient groups, with particularly notable differences in hematological markers. The strong predictive value of hemoglobin levels (SHAP value 0.42) for CKD aligns with established pathophysiological mechanisms and supports its inclusion as a key feature in predictive models [9]. Similar correlations were observed between age and cognitive decline in dementia prediction (SHAP value 0.45), supporting the model's biological plausibility.

In developing nations, particularly in South Asia, the implementation of ML models presents both opportunities and challenges [10]. Our study's balanced distribution of cases across disease categories (19.2-21.2%) helped mitigate potential bias, though the single-center nature may limit generalizability. While our sample size of 270 patients exceeded the minimum calculated requirement, larger multi-center studies would be valuable for external validation [11,12].

Regional studies have demonstrated comparable findings, particularly in South Asian populations [13,14]. The observed variation in model performance across different diseases provides insights into the strengths and limitations of different algorithms. Random forest demonstrated superior performance in conditions with well-defined biochemical markers, while logistic regression showed advantages in diseases with more complex, multifactorial presentations [15,16].

Model interpretability remains crucial, particularly in healthcare settings where understanding the basis of predictions is essential for clinical decision-making [17,18]. Our approach of combining ChatGPT's natural language processing capabilities with traditional ML algorithms offers a potential framework for developing more interpretable models while maintaining predictive accuracy.

The selection of specific ML algorithms for different diseases reflected their inherent characteristics and the nature of predictive variables. Random forest demonstrated superior performance for CKD and heart disease through its capacity to handle non-linear relationships between multiple laboratory parameters and manage complex interaction effects. Logistic regression proved more effective for TB and dementia, where categorical clinical features predominate and interpretability is crucial for clinical decision-making. The clinical utility of these models lies in their ability to identify high-risk patients using routine parameters before definitive diagnostic criteria are met, potentially enabling earlier intervention and more efficient resource allocation in screening programs.

These models offer several practical benefits in clinical settings. They enhance risk stratification using routine parameters and improve screening efficiency in resource-limited environments by identifying subtle parameter combinations that might be overlooked in standard clinical assessment. The CKD model demonstrated the ability to identify high-risk patients using routine blood parameters months before significant eGFR decline, potentially enabling earlier intervention. Similarly, the heart disease model's integration of multiple routine parameters showed promise in identifying patients requiring urgent cardiac evaluation, potentially reducing diagnostic delays.

Several limitations of our study require acknowledgment. First, the single-center design may limit external validity. Second, while our sample size was statistically adequate, larger cohorts would enable more robust subgroup analyses. Third, the cross-sectional nature of our data collection may not fully capture disease progression patterns [19]. Fourth, while ChatGPT integration showed promising results, the rapid evolution of language models necessitates ongoing validation of their applications in clinical settings [20].

A significant consideration in our methodology was the careful separation of predictive variables from diagnostic criteria. We addressed this challenge by developing two-stage models and conducting temporal validation to ensure true predictive capability rather than mere diagnostic confirmation. Future studies should focus on prospective validation and the integration of time-series data to further strengthen the predictive value of these models.

Future research directions should focus on external validation through multi-center studies with larger cohorts, longitudinal assessment of model performance over time, integration of genomic and environmental data to enhance prediction accuracy, development of standardized protocols for language model integration in clinical AI applications, and evaluation of implementation strategies in resource-limited settings.

## Conclusions

The integration of ChatGPT-assisted ML approaches in chronic disease prediction demonstrates significant potential for enhancing diagnostic capabilities in clinical settings. Our findings establish that combining AI with traditional clinical parameters can create robust predictive models across multiple chronic conditions, which is particularly valuable for resource-limited healthcare environments. The successful implementation of these models, with their high diagnostic accuracy and interpretability, suggests a promising pathway for improving early disease detection and patient outcomes in South Asian healthcare settings.

Future implementation of these AI systems in clinical practice should focus on three key areas: external validation through multi-center studies, development of standardized integration protocols, and comprehensive healthcare provider training programs. While our results support the feasibility of AI-assisted diagnostic tools, successful clinical integration will require careful consideration of practical challenges, including resource availability, system compatibility, and cost-effectiveness. This balanced approach to technological advancement in healthcare promises to enhance diagnostic capabilities while maintaining the essential role of clinical expertise in patient care.
